# Global DNA methylation pattern involved in the modulation of differentiation potential of adipogenic and myogenic precursors in skeletal muscle of pigs

**DOI:** 10.1186/s13287-020-02053-3

**Published:** 2020-12-11

**Authors:** Xin Zhang, Wenjuan Sun, Linjuan He, Liqi Wang, Kai Qiu, Jingdong Yin

**Affiliations:** 1 State Key Laboratory of Animal Nutrition, College of Animal Science and Technology, China Agricultural University, Beijing, 100193 China; 2 State Key Laboratory of Agrobiotechnology, College of Biological Sciences, China Agricultural University, Beijing, 100193 China

**Keywords:** Adipogenic precursors, Myogenic precursors, DNA methylation, CACNA2D2-JNK/MAPK axis, Cell fate commitment, Skeletal muscle homeostasis

## Abstract

**Background:**

Skeletal muscle is a complex and heterogeneous tissue accounting for approximately 40% of body weight. Excessive ectopic lipid accumulation in the muscle fascicle would undermine the integrity of skeletal muscle in humans but endow muscle with marbling-related characteristics in farm animals. Therefore, the balance of myogenesis and adipogenesis is of great significance for skeletal muscle homeostasis. Significant DNA methylation occurs during myogenesis and adipogenesis; however, DNA methylation pattern of myogenic and adipogenic precursors derived from skeletal muscle remains unknown yet.

**Methods:**

In this study, reduced representation bisulfite sequencing was performed to analyze genome-wide DNA methylation of adipogenic and myogenic precursors derived from the skeletal muscle of neonatal pigs. Integrated analysis of DNA methylation and transcription profiles was further conducted. Based on the results of pathway enrichment analysis, myogenic precursors were transfected with CACNA2D2-overexpression plasmids to explore the function of CACNA2D2 in myogenic differentiation.

**Results:**

As a result, 11,361 differentially methylated regions mainly located in intergenic region and introns were identified. Furthermore, 153 genes with different DNA methylation and gene expression level between adipogenic and myogenic precursors were characterized. Subsequently, pathway enrichment analysis revealed that DNA methylation programing was involved in the regulation of adipogenic and myogenic differentiation potential through mediating the crosstalk among pathways including focal adhesion, regulation of actin cytoskeleton, MAPK signaling pathway, and calcium signaling pathway. In particular, we characterized a new role of CACNA2D2 in inhibiting myogenic differentiation by suppressing JNK/MAPK signaling pathway.

**Conclusions:**

This study depicted a comprehensive landmark of DNA methylome of skeletal muscle-derived myogenic and adipogenic precursors, highlighted the critical role of CACNA2D2 in regulating myogenic differentiation, and illustrated the possible regulatory ways of DNA methylation on cell fate commitment and skeletal muscle homeostasis.

**Supplementary information:**

The online version contains supplementary material available at 10.1186/s13287-020-02053-3.

## Background

Pigs, serving as a major animal protein source, provide more than 100,000,000 t of pork annually for human consumption. Undergone intensive selection for lean meat percentage for decades, pigs are distinguished for their muscle development potential and meat production efficiency. In skeletal muscle, myocytes and adipocytes are both derived from mesenchymal stem cells (MSCs) [1–3]. Myogenesis is mediated by extrinsic signals derived from a variety of cells existing in the niche of muscle stem cells [4–6], and occurs competitively with adipogenesis during the periods of embryonic and neonatal development [7]. Adipogenesis and subsequent lipid deposition in skeletal muscle constitute intramuscular fat mainly stored in adipocyte dispersed in the muscle fascicle. Excessive intramuscular fat accumulation not only undermines skeletal muscle functions and metabolic homeostasis in humans [8, 9] but also endows muscle with marbling-related characteristics of economic value in farm animals [10]. Therefore, it is of great significance to elucidate mechanisms governing the balance of myogenesis and adipogenesis in skeletal muscle.

DNA methylation plays an important role in various biological processes, including cell lineage specification and cell differentiation [11, 12]. For example, demethylation in the promoter of zinc-finger protein 423, a transcriptional regulator for preadipocyte determination [13], promotes adipogenic differentiation of obesogenic fetal tissue [14] and murine 3T3-L1 cells [15]. Accordingly, silencing DNA methyltransferase 1 (DNMT1) accelerated adipocyte differentiation and lipid accumulation accompanied with an abnormal early induction of adipocyte-specific genes, such as glucose transporter 4, fatty acid-binding protein 4, and peroxisome proliferator-activated receptor gamma (PPARγ) [16]. Moreover, it has been shown that remarkable hyper-methylation takes place during myogenic lineage commitment of murine embryonic stem cells (ESCs) and myogenic differentiation of human myoblasts [17, 18] accompanied by the DNA demethylation of myogenic factor 5 (Myf5) [17]. Remarkably, demethylation occurred in the promoter of myogenin (MyoG), key transcriptional factor involved in myogenic differentiation, at the onset of C2C12 myoblast differentiation and in embryonic mouse muscle compared with non-muscle tissues, which further highlighted the regulatory role of DNA methylation in myogenesis [19, 20]. Nevertheless, approximately 3.7-folds more methylation changes and increased DNMT1 expression were also observed during myogenesis from obese versus non-obese subjects [21]. Until now, however, the role of DNA methylation in maintaining balance of myogenesis and adipogenesis in skeletal muscle remains unclear and merits further investigation.

The mitogen-activated protein kinase (MAPK) signaling pathway, a phosphorylation kinase signaling cascade, plays a vital role in regulating cell division and differentiation [22, 23]. The c-Jun amino-terminal kinases (JNK)/MAPK signaling pathway regulated muscle remodeling through myostatin/SMAD signaling, and the suppression of JNK/MAPK resulted in smaller and more oxidative muscle fibers [24]. Besides, mounting studies showed that JNK/MAPK pathway positively regulated adipogenesis [25, 26]. However, the role of JNK/MAPK signaling in cell differentiation potential of myogenic and adipogenic precursors in skeletal muscle still remains unclear.

In the present study, myogenic and adipogenic precursors derived from the skeletal muscle of neonatal pigs were subjected to reduced representation bisulfite sequencing (RRBS) and RNA-seq analysis to explore the role of DNA methylation in regulating the balance of myogenesis and adipogenesis in skeletal muscle.

## Materials and methods

### Cell isolation, culture, and differentiation

This study was approved by the Institutional Animal Care and Use Committee (CAU20161110-2) of China Agricultural University. Adipogenic and myogenic precursors were isolated from the skeletal muscle of neonatal pigs using a preplate technique as described previously [27, 28]. Briefly, skeletal muscle was minced and digested by 0.17% protease (Sigma-Aldrich, USA) at 37 °C for 1 h, and 0.15% collagenase-type XI solution (Sigma-Aldrich, USA) for 1 h in turn. Completely digested muscles were then filtered through a 40-μm nylon cell strainer. Isolated cells were plated for 2 h in growth medium (GM, DMEM supplemented with 10% FBS (Gibco, USA), 2 mM glutamine, 1% penicillin-streptomycin, and 5 ng/ml basic fibroblast growth factor) on collagen I-coated dishes at 37 °C in a 5% CO_2_ atmosphere. Then, non-adherent cells were removed to another dish and further collected after 72-h adhering. The first (0–2 h) and second (2–74 h) sets of adherent cells were adipogenic and myogenic precursors, respectively.

To induce myogenic differentiation, cells at 80–90% confluence were switched to differentiation medium (DM) containing DMEM and 2% horse serum (ThermoFisher, USA). To inhibit the activity of JNK/MAPK signaling pathway, myogenic precursors were treated with 10 μM JNK-specific inhibitor SP600125 (MedChemExpress, USA) [29, 30] during myogenic differentiation. To induce adipogenic differentiation, cells were treated with adipogenic DM containing 10% FBS, 1 μM dexamethasone, 0.5 mM 1-methyl-3-isobutylmethyl-xanthine, and 10 μg/ml insulin. After 3 days, medium was replaced with maintenance medium (DMEM containing 10% FBS and 10 μg/ml insulin) for 6 days.

### RNA extraction and qRT-PCR assays

Total RNA was extracted from cells using RNAiso Plus (Takara, China) according to the manufacturer’s instruction. qRT-PCR assays were conducted as previously described [27]. GAPDH was used as an internal control. Data were analyzed by relative expression in 2^−ΔΔCt^ method [31]. Primers used for qRT-PCR were shown in Additional file 3: Table S1.

### Western blotting

Protein samples were extracted in RIPA lysis buffer (Huaxingbio, China) with protease inhibitor cocktail (Roche, Switzerland) and phosphatase inhibitor cocktail (Roche, Switzerland). Protein concentration was determined using the BCA Protein Assay Kit (Huaxingbio, China). Equal amounts of protein samples were separated by 10% SDS-PAGE and transferred to polyvinylidene difluoride (PVDF) membrane. PVDF membrane was blocked with 5% bovine serum albumin (BSA) for 1 h at room temperature (RT) and then incubated with the following primary antibodies: antibodies for ERK (#4695), p-ERK (Thr202/Tyr204, #4370), JNK (#9252), p-JNK (Thr183/Tyr185, #4668), p38 MAPK (#8690), p-p38 MAPK (Thr180/Tyr182, #4511), c-Jun (#9165), p-c-Jun (Ser63, #3270), and GAPDH (#2118) were purchased from Cell Signaling Technology. Antibody for MyoG (ab124800) was purchased from Abcam. Antibody for CACNA2D2 (CSB-PA889145ESR2HU) was obtained from Cusabio (http://www.cusabio.cn/). Following incubated with DyLight 800-labeled secondary antibodies and detected with the Odyssey Clx (LiCor Biosciences, USA), gray analysis of blots was performed using ImageJ (National Institutes of Health, Bethesda, USA) software.

### Oil red O staining

After adipogenic induction, cells were fixed with 10% formalin at RT for 1 h and stained with a filtered Oil Red O (Sigma-Aldrich, USA) working solution for 10 min. Stained cells were washed with distilled water and photographed using an inverted microscope. To quantify triglyceride accumulation, Oil Red O-stained lipids were eluted in 100% isopropanol, and the optical density (OD) was measured at 520 nm.

### Immunofluorescence

Cells were fixed with 4% paraformaldehyde for 30 min at RT and quickly washed twice with PBS before permeabilization with 0.2% Triton X-100 in PBS for 10 min. Cells were then blocked in blocking solution (5% BSA in PBS) for 1.5 h and incubated with primary antibodies against Myosin (1:300, cat. M4276, Sigma-Aldrich, USA) and p-c-Jun (1:300, cat. #3270, Cell Signaling Technology, USA) at 4 °C overnight. After three washed in PBS, cells were incubated with secondary antibodies (ZSGB-BIO, China) in blocking buffer for 1 h at RT. Finally, nuclei were counterstained with DAPI (1:1000 in PBS) and fluorescence was visualized using Olympus fluorescence microscope.

### Plasmids and transfection

CACNA2D2 cDNA fragments were cloned into a pcDNA3.1 vector (with EGFP labeling) between EcoRІ and BamHІ sites. Plasmid transfection was performed using Lipofectamine™ 3000 Transfection Reagent (Invitrogen, USA) according to the manufacturer’s instruction. After 24 h, transfected myogenic precursors were switched into myogenic DM.

### Reduced representation bisulfite libraries construction and sequencing

Myogenic and adipogenic precursors in GM rather than DM were collected to analyze the DNA methylation pattern using RRBS. DNA samples extracted from myogenic and adipogenic precursors of three pigs were included in this study (*n* = 3). Experimental and computational procedures were carried out as previously described [32, 33] with some modifications. Briefly, 600 μg of genomic DNA sample was digested with MspI (New England Biolabs, USA). The digested product was subsequently purified with the AMPureXP (Becman, USA), end repaired, 3′-end-adenylated, and adapter-ligated using NEXTflex™ Bisulfite-seq Kit (Illumina, USA). Fragments with 225–290 bp size were collected by gel electrophoresis, purified, and bisulfite treated using EZ DNA Methylation-Gold Kit (Zymo research, USA) under the manufacture instructions. The bisulfite converted DNA was then PCR amplified, purified by Agencourt AMPure XP Beads (Beckman, USA), and validated using Agilent 2100 Bioanalyzer (Agilent Technologies, USA). Libraries were sequenced as 126-bp paired-end sequencing reads using Illumina Hiseq 2500.

### Analysis and annotation of differentially methylated regions (DMRs)

Differential methylation was analyzed at both the single CpG and regional levels. Sequenced reads were trimmed and mapped to pig reference genome (Sscrofa10.2) using Bismark [34]. Only autosome CpGs (sequence depth > 10) common to all six samples were included in all subsequent analyses. Raw read counts were normalized using the Estimate Size Factors function in the DESeq package in R statistical program [35]. For each window, paired *t* test was conducted to analyze differences between adipogenic and myogenic precursors from the same pig using normalized read counts. *P* values were adjusted by the Benjamini-Hochberg method. A methylated region was considered significant if the difference between samples was at least 3%, and the corrected *P* value (FDR) was less than 0.05.

DMRs were annotated with ensemble genes, transcripts, promoters, CpG islands (CGIs), and repeat mask. Genomic locations of these genomic features were obtained from the UCSC pig reference genome (Sscrofa10.2). As described in previous study [36], we defined the genomic region from − 2000 to the transcription start site (TSS) as the promoter region, and from TSS to transcription termination site as the gene body region. DNA regions greater than 200 bp with a CG content equal to or greater than 50% and an observed CpG/expected CpG excess of 0.6 were defined as CGIs. The sequences 2 kb up/downstream from CGIs were termed as CpG island shores [33]. All statistical analysis was performed using R package.

### Motifs discovery of DMRs

Hyper- or hypo-methylated regions were tested for motif discovery and transcription factor binding sites analysis. DMRs, used as input sequences in multi-FASTA format, were subjected to MEME for motifs analysis (http://meme.nbcr.net/meme/). Subsequently, identified motifs were subjected to a motif comparison tool, Tomtom (http://meme.nbcr.net/meme/cgi-bin/tomtom.cgi), for comparison with known motifs in databases. System default parameters were used for MEME and Tomtom.

### Gene expression profile of myogenic and adipogenic precursors

RNA-seq dataset of myogenic and adipogenic precursors derived from skeletal muscle of neonatal pigs reported in our previous study was mined here [28]. FDR < 0.05 and fold change > 2 or < 0.5 were set as the threshold for differentially expressed genes (DEGs) between adipogenic and myogenic precursors.

### Functional enrichment and network analysis

Kyoto Encyclopedia of Genes and Genomes (KEGG) pathway enrichment analysis based on DAVID bioinformatics resources was performed to predict the function of DMR-related genes and genes with different DNA methylation and expression levels. Cut off criteria were set with *P* value < 0.05 or Benjamini-Hochberg FDR < 0.05 for KEGG enrichment analysis as shown in figure legends. Cytoscape software (v.3.5.1; Cytoscape Consortium, USA) was applied to visualize KEGG enrichment results.

### Statistical analysis

Data organization, scientific graphing, and statistical analysis were conducted by Microsoft Excel (Redmond, USA), GraphPad Prism (v.6; USA), and SAS (v.9.2; SAS Institute, USA), respectively. Data were presented as means ± SEM. Statistical differences were determined by the unpaired 2-tailed Student’s *t* test or one-way ANOVA analyses followed by post hoc Tukey’s tests as indicated in figure legends. A value of *P* < 0.05 was considered significant.

## Results

### Identification of adipogenic and myogenic precursors

Adipogenic and myogenic precursors were isolated from skeletal muscle of neonatal pigs with distinct adhesion characteristics to collagen I-coated dishes. Upon myogenic differentiation, multi-nuclei myotubes were present in myogenic precursors (Additional file 1: Figure S1b). Consistent with myotube formation, higher expression levels of myogenic-specific genes, such as myoblast determination protein 1 (MyoD1), MyoG, myocyte enhancer factor 2C (MEF2C), and Myf5, were also observed in myogenic precursors as shown by RNA-seq data (Additional file 1: Figure S1d), while myotube formation was absent in adipogenic precursors upon myogenic introduction (Additional file 1: Figure S1b). RNA-seq analysis showed that adipogenic precursors expressed higher mRNA levels of adipocyte-specific genes than myogenic precursors, such as CCAAT/enhancer binding protein alpha (C/EBPα), PPARγ, and platelet-derived growth factor receptor alpha (PDGFRα) (Additional file 1: Figure S1d), evidenced by the lipid accumulation under adipogenic induction as shown by Oil Red O staining (Additional file 1: Figure S1c, e). While, little or no adipogenic potential was evident in myogenic precursors upon adipogenic induction (Additional file 1: Figure S1c, d and e). These results demonstrated that adipogenic and myogenic precursors collected in this study possessed distinct differentiation potential.

### Global DNA methylation pattern of adipogenic and myogenic precursors

Adipogenic and myogenic precursors (*n* = 3) were subjected to RRBS to quantify DNA methylation. After filtration of raw data, 20.5 G clean bases were obtained, with the number of sequence bases range from 3.2 to 3.6 G single paired reads for all six samples, and Q20 was above 95%. About 50% clean reads of each sample were mapped to *Sus scrofa* genome (Sscrofa10.2), and the bisulfate conversion ratio was above 99.5% (Additional file 4: Table S2).

**Fig. 1:**
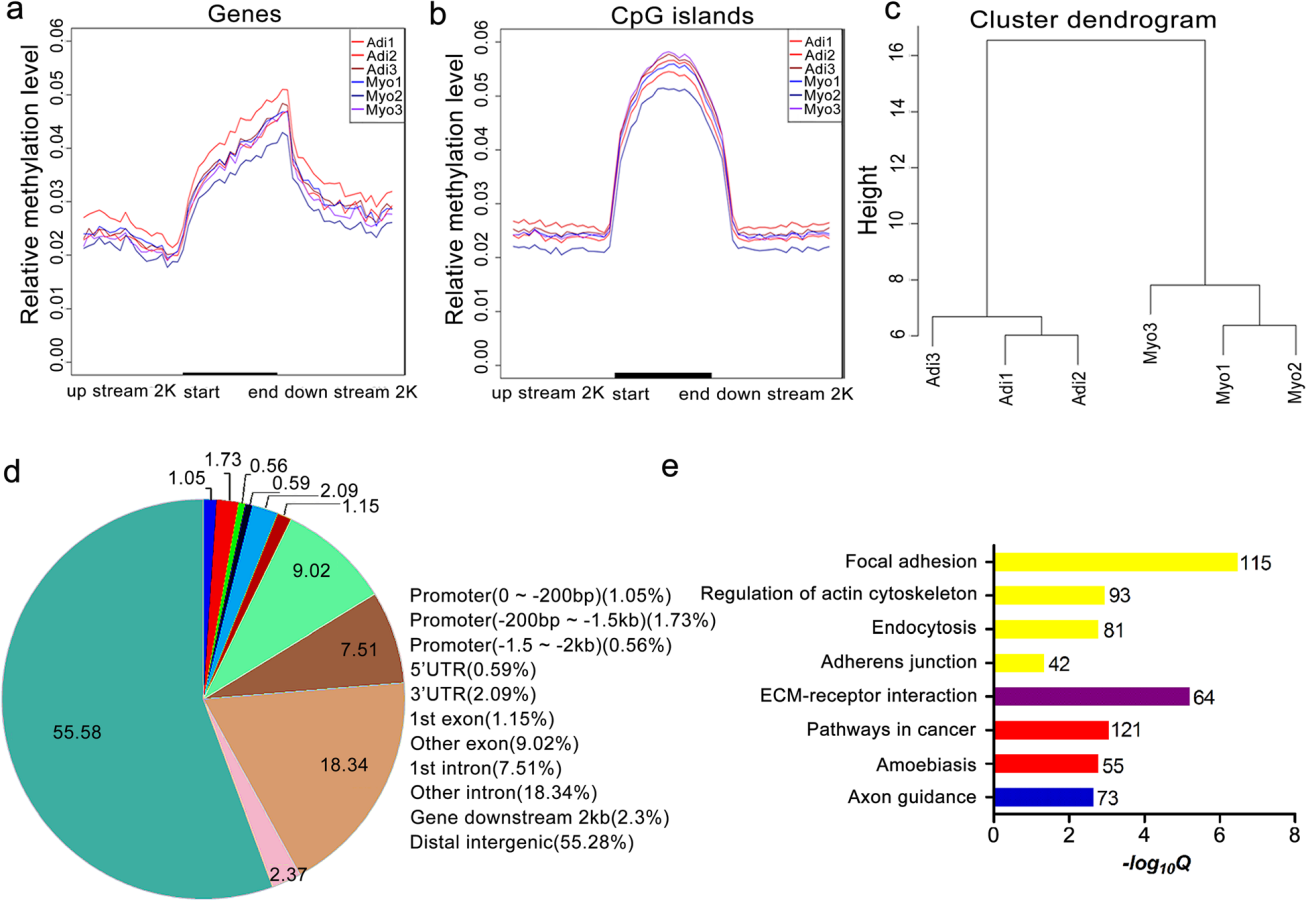
Global DNA methylation pattern and differentially methylated region (DMR) identification of adipogenic and myogenic precursors. **a** DNA methylation level across genes in all six samples. **b** DNA methylation level across CpG islands in all six samples. **c** Hierarchical clustering of adipogenic and myogenic precursors using DNA methylation patterns present at DMRs. **d** Distribution of DMRs in different genomic features. **e** Enriched KEGG pathways (*Q* < 0.05) based on all DMR-related genes. The number on the right of column represents the number of genes enriched in each pathway. Adi, adipogenic precursors; Myo, myogenic precursors

Comprehensive analysis was performed to detect the distribution of DNA methylation in the 2.0 kb region upstream/downstream of TSS and gene body. DNA methylation levels decreased sharply at TSS and increased dramatically towards gene body (Fig. 1a). Considering the great significance of CGIs in gene expression regulation [12], we analyzed the methylation status of CGIs and CGI shores and found that DNA methylation level increased sharply towards CGIs (Fig. 1b). However, there was no significant difference in methylation level between adipogenic and myogenic precursors regardless of gene body or CGIs (Fig. 1a, b).

### Identification of DMRs

In this study, we identified 11,361 DMRs between adipogenic and myogenic precursors, among them 5372 (47.3%) were hyper-methylated in myogenic precursors relative to adipogenic precursors, while 5989 (52.7%) DMRs were hypo-methylated in myogenic precursors. Importantly, DMR-based hierarchical clustering for six samples showed a clear difference between adipogenic and myogenic precursors (Fig. 1c). Next, we compared the genomic features and DMRs. The majority of DMRs (55.28%) appeared in distal intergenic region. Meanwhile, the percentage of DMRs in intron (first intron, 7.51%; other intron, 18.34%) was higher than that in exon (first exon, 1.15%; other exon, 9.02%), and 3.34% DMRs were distributed along promoter sequences (0 to about − 200 bp, 1.05%; − 200 bp to about − 1.5 kb, 1.73%; − 1.5 to about 2.0 kb, 0.56%) (Fig. 1d).

To explore the relationship between DNA methylation and cell fate commitment of myogenic and adipogenic precursors, KEGG pathway analysis of 5333 DMR-related genes were conducted. A total of eight significantly enriched KEGG pathways (*q* < 0.05) were identified, and among them focal adhesion (*q* = 2.97E− 07, 115 genes involved), regulation of actin cytoskeleton (*q* = 9.97E− 04, 93 genes involved), endocytosis (*q* = 1.50E− 03, 81 genes involved), and adherens junction (*q* = 4.21E− 02, 42 genes involved) belong to cellular process. Furthermore, ECM-receptor interaction (*q* = 5.55E− 06, 64 genes involved) and axon guidance (*q* = 2.01E− 03, 73 genes involved) were also enriched along with pathways in cancer (*q* = 7.94E− 04, 121 genes) and amoebiasis (*q* = 1.50E− 03, 55 genes involved) associated with human diseases (Fig. 1e).

### Potential impact of DNA methylation on gene expression and transcriptional factors binding

**Fig. 2:**
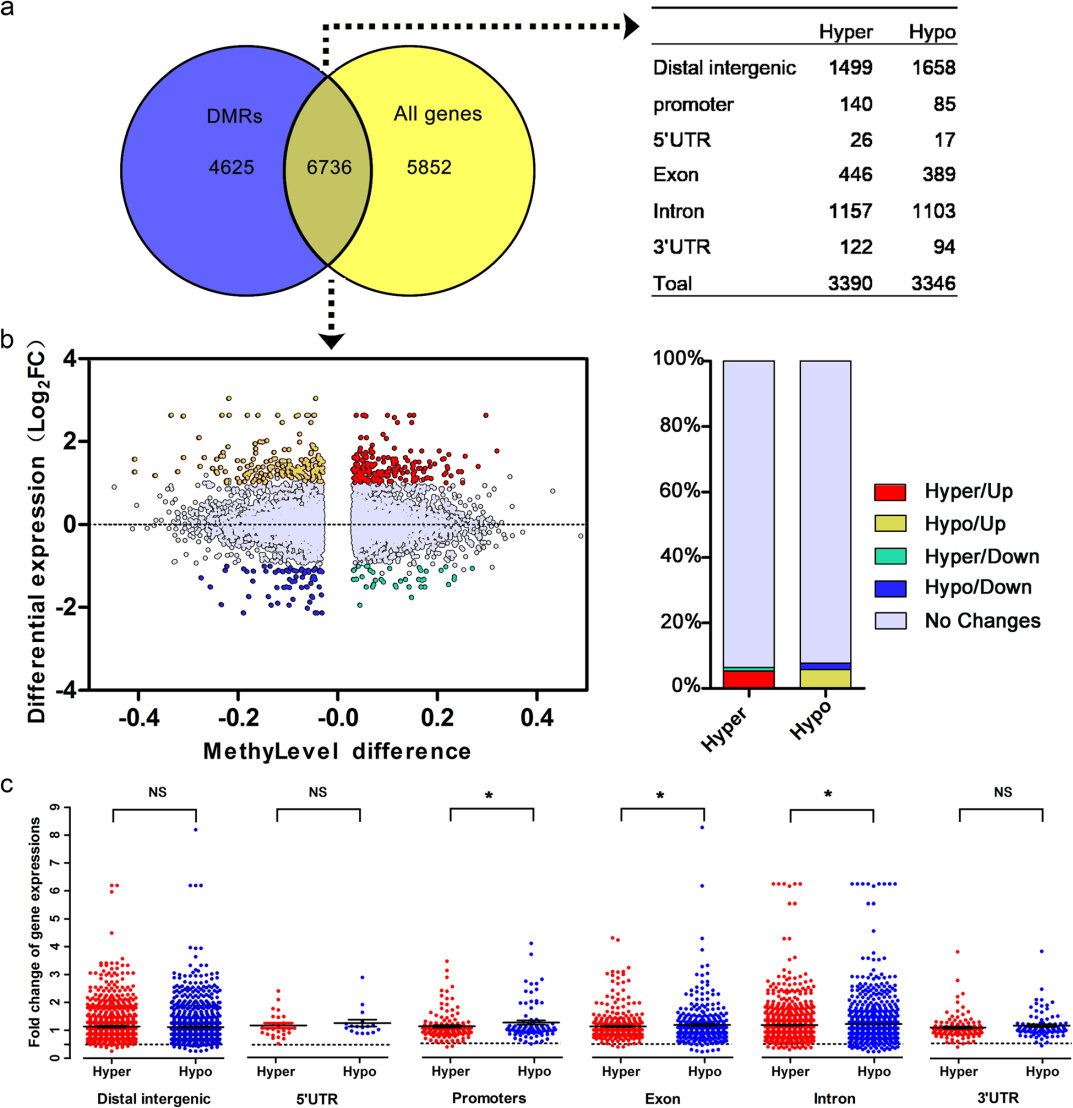
Correlation of gene expression and differentially methylated regions (DMRs) between adipogenic and myogenic precursors. **a** The links of DMRs and detected genes in adipogenic and myogenic precursors. **b** Scatterplot of DMR-linked genes, integrating significant change in DNA methylation (*x*-axis) and gene expression (*y*-axis). DMRs with ±2-fold changes are yellow, red, blue, and green colored dependent on the methylation and expression status, whereas DMRs with no changes in gene expression are gray colored. Column graph (right) indicating the percentage of hypo- and hyper-methylated DMRs showing up or down gene regulation in myogenic precursors compared with that in adipogenic precursors. **c** Dot plots of gene expression fold changes between adipogenic and myogenic precursors for hyper-methylated (red circles) and hypo-methylated (blue circles) regions. Black horizontal lines in the boxplots indicated median fold change.^*^P < 0.05, NS, no significance

To explore the interplay between DNA methylation and gene expression patterns of adipogenic and myogenic precursors, we conducted an integrated analysis of DNA methylation and gene expression data. A total of 12,588 genes were detected by RNA-seq analysis, and 11,361 DMRs were linked to the closest annotated coding gene (Fig. 2a). Results showed that 6376 DMRs, among which 3390 were hyper-methylated and 3346 were hypo-methylated in myogenic precursors relative to adipogenic precursors, were assigned to 5333 genes, and DMRs distributed across the distal intergenic region, promoter, and gene body (Fig. 2a). As shown in Fig. 2b, 181 hyper-methylated and 194 hypo-methylated DMRs overlapped with upregulated genes (fold change > 2 and FDR < 0.05) shown as red and orange dots, respectively. In addition, 37 hyper-methylated and 65 hypo-methylated DMRs overlapped with downregulated genes (fold change < 0.5 and FDR < 0.05) shown as green and blue dots, respectively. Among DMR-related genes, the number of upregulated genes exceeded downregulated genes. Next, the fold changes in gene expression of myogenic precursors compared with adipogenic precursors were plotted for hyper- and hypo-methylated genes separately (shown as red and blue dots, respectively) for each of the six categories (Fig. 2c). As shown in Fig. 2c, the expression fold changes of hypo-methylated genes were significantly greater than those of hyper-methylated genes in the categories exon, intron, and promoters, while no significant differences were observed on expression levels of genes between hypo- and hyper-methylated distributed in distal intergenic region, 5′-UTR, and 3′-UTR, respectively (Fig. 2c). Additionally, among the DEGs detected by RNA-seq analysis, 153 DEGs were also differentially methylated between adipogenic and myogenic precursors, namely co-different genes (Additional file 5: Table S3).

**Fig. 3:**
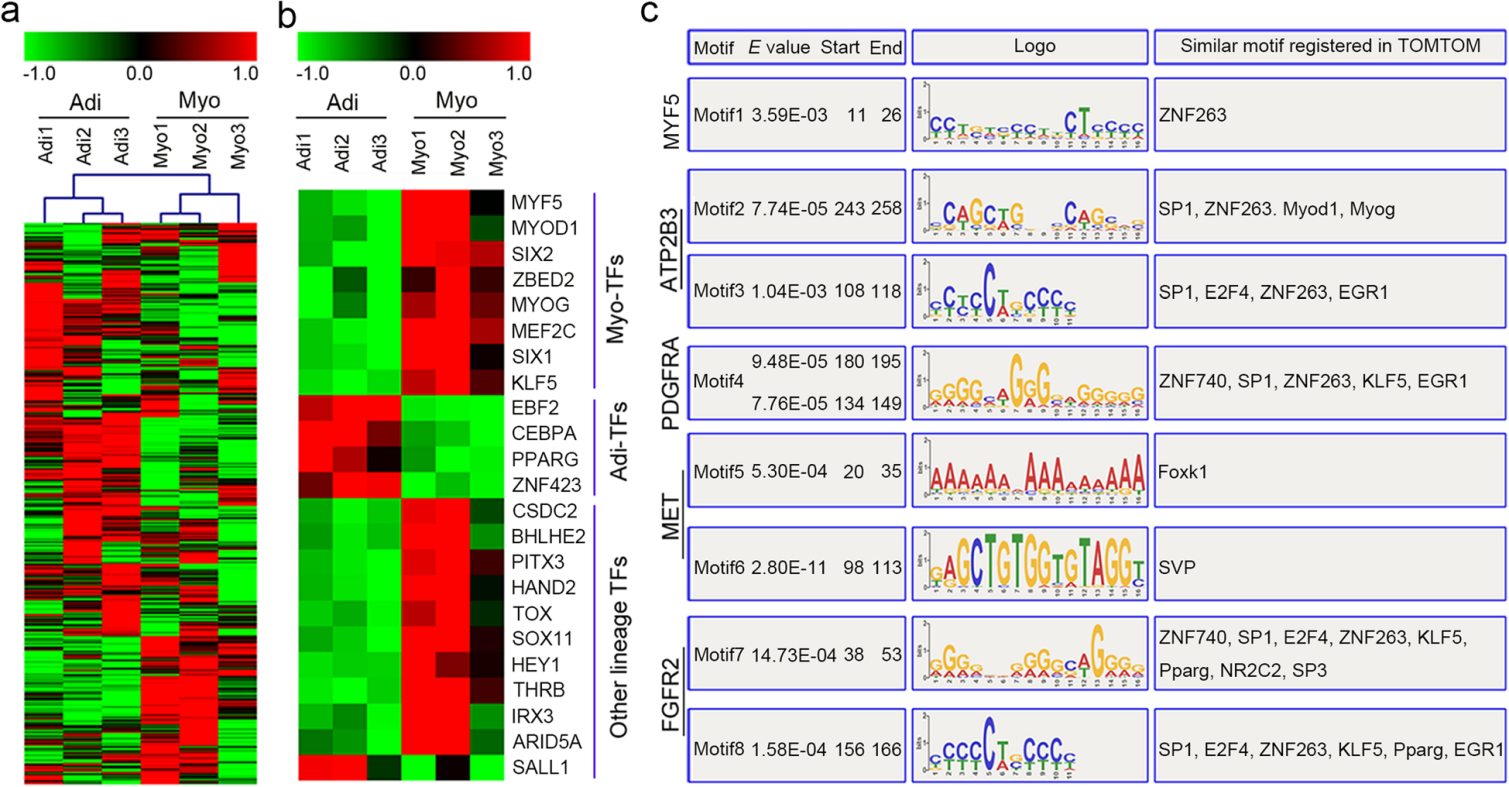
Transcription factor levels and methylation status of their binding sites in adipogenic and myogenic precursors. **a** All transcription factor (TF)-based hierarchical clustering. **b** Differentially expressed TF-based hierarchical clustering and their relationship with adipogenic and myogenic differentiation. **c** DNA-binding motifs of TFs that identified highly similar with DMRs (*P* < 0.01) located in co-different genes. Adi, adipogenic precursors; Myo, myogenic precursors; TFs, transcription factors

Transcription factors (TFs) that may act as targets of DNA methylation were also identified. Briefly, a total of 534 TFs were identified by RNA-seq (Fig. 3a). Among them, 23 TFs were differentially expressed between adipogenic and myogenic precursors (Fig. 3b). Briefly, 18 TFs were highly expressed in myogenic precursors, while five TFs were highly expressed in adipogenic precursors (Additional file 6: Table S4). Notably, 12 differentially expressed TFs were well-known to be implicated with adipogenic and myogenic differentiation, among them myogenic-specific TFs, such as MYF5, MYOD1, MYOG, MEF2C, SIX1, SIX2, ZBED2, and KLF5 were highly expressed in myogenic precursors, while adipogenic-specific TFs, including C/EBPα, PPARγ, ZNF423, and EBF2 were highly expressed in adipogenic precursors (Fig. 3b).

DMRs distributed in promoters and gene bodies were then subjected to MEME analysis to enrich motifs, and then the TFs combining the top 20 motif were identified by screening Tomtom database (Additional file 7: Table S5). Tomtom analysis revealed that DNA methylation may influence the binding of TFs with co-different genes. For example, the DNA-binding motif of ZNF263 was similar to the motif enriched in MYF5-related DMRs (Fig. 3c). Additionally, DNA-binding motifs of SP1, ZNF263, MYOD1, MYOG, E2F4, and EGR1 were also similar to motifs enriched in ATP2B3-related DMRs (Fig. 3c). In short, we can reasonably deduce that differentially expressed TFs and DMRs where TFs binding on target genes might be involved in coordinately regulating distinct fate commitment of myogenic and adipogenic precursors in skeletal muscle.

### Functional annotation of co-different genes

**Fig. 4:**
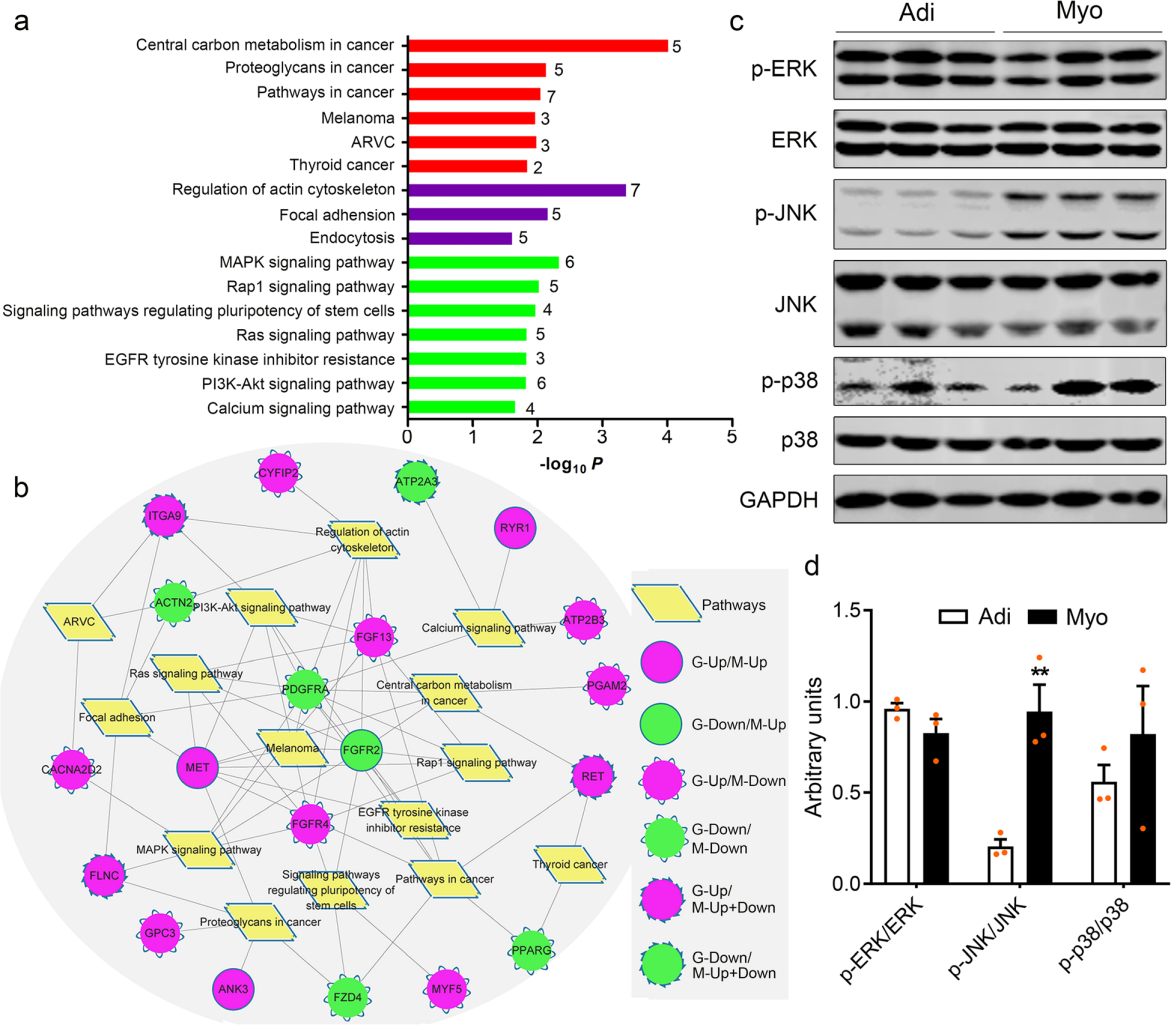
Functional annotation of co-different genes and differences in MAPK signaling of adipogenic and myogenic precursors. **a** The significant KEGG pathways (*P* < 0.05) enriched with co-different genes between adipogenic and myogenic precursors. Red, purple, and green columns represent pathways related to human diseases, cellular process, and extracellular environmental processing, respectively. The number on the right of column represents the number of genes enriched in each pathway. **b** Cluster of KEGG pathways and co-different genes. G, gene expression level; M, DNA methylation level. **c** The expression of MAPK signaling key factors (ERK, JNK, and p38) and their phosphorylated forms (p-ERK, p-JNK, and p-p38) was detected by western blotting between adipogenic and myogenic precursors. **d** Relative expression level in **c** was calculated. GAPDH was used as the internal control. Data were presented as means ± SEM (*n* = 3). The statistical significance of difference between two means was calculated using *t* test, ^**^P < 0.01

To enrich the possible core signaling pathways involved in the distinct determination of adipogenic and myogenic precursors, 153 co-different genes were subjected to KEGG analysis, and a total of 16 significantly enriched pathways (*P* < 0.05) were identified (Fig. 4a). Besides six signaling pathways implicated with human diseases, there were 10 following signaling pathways mediating the cell proliferation and differentiation. Regulation of actin cytoskeleton (*P* = 0.0004, 7 genes involved), focal adhesion (*P* = 0.0065, 5 genes involved), and endocytosis (*P* = 0.023, 5 genes involved) were clustered into cellular process; MAPK signaling pathway (*P* = 0.004, 6 genes involved), Rap1 signaling pathway (*P* = 0.009, 5 genes involved), signaling pathways regulating pluripotency of stem cells (*P* = 0.010, 4 genes involved), Ras signaling pathway (*P* = 0.014, 5 genes involved), EGFR tyrosine kinase inhibitor resistance (*P* = 0.014, 3 genes involved), PI3K-Akt signaling pathway (*P* = 0.014, 6 genes involved), and calcium signaling pathway (*P* = 0.021, 4 genes involved) belonged to extracellular environmental processing. To understand the mutual roles of these co-different genes in the distinct potential of precursors, Cytoscape (v3.2.1) was applied to depict a putative molecular network embracing 16 enriched pathways and co-different genes known to be involved in (Fig. 4b). A total of 20 co-different genes and, particularly, fibroblast growth factor 13 (FGF13); fibroblast growth factor receptor (FGFR2 and FGFR4); MET proto-oncogene, receptor tyrosine kinase (MET); PDGFRα; and integrin subunit alpha 9 (ITGA9) acted as hubs to be involved in the above 16 pathways, implying the key role of these genes in commitment or differentiation of adipogenic and myogenic precursors.

### Positive role of JNK/MAPK signaling pathway in myogenic differentiation

Mammals express at least three classical groups of MAPKs, extracellular signal-related kinases (ERK)-1/2, Jun amino-terminal kinases (JNK1/2/3), and p38 proteins (p38alpha/beta/gamma/delta). Particularly, in the present study, only the phosphorylation of JNK was extremely enhanced in myogenic precursors relative to adipogenic precursors (*P* < 0.01) (Fig. 4c, d), implying the key role of the JNK/MAPK signaling pathway in distinct potential of myogenic and adipogenic precursors.

**Fig. 5:**
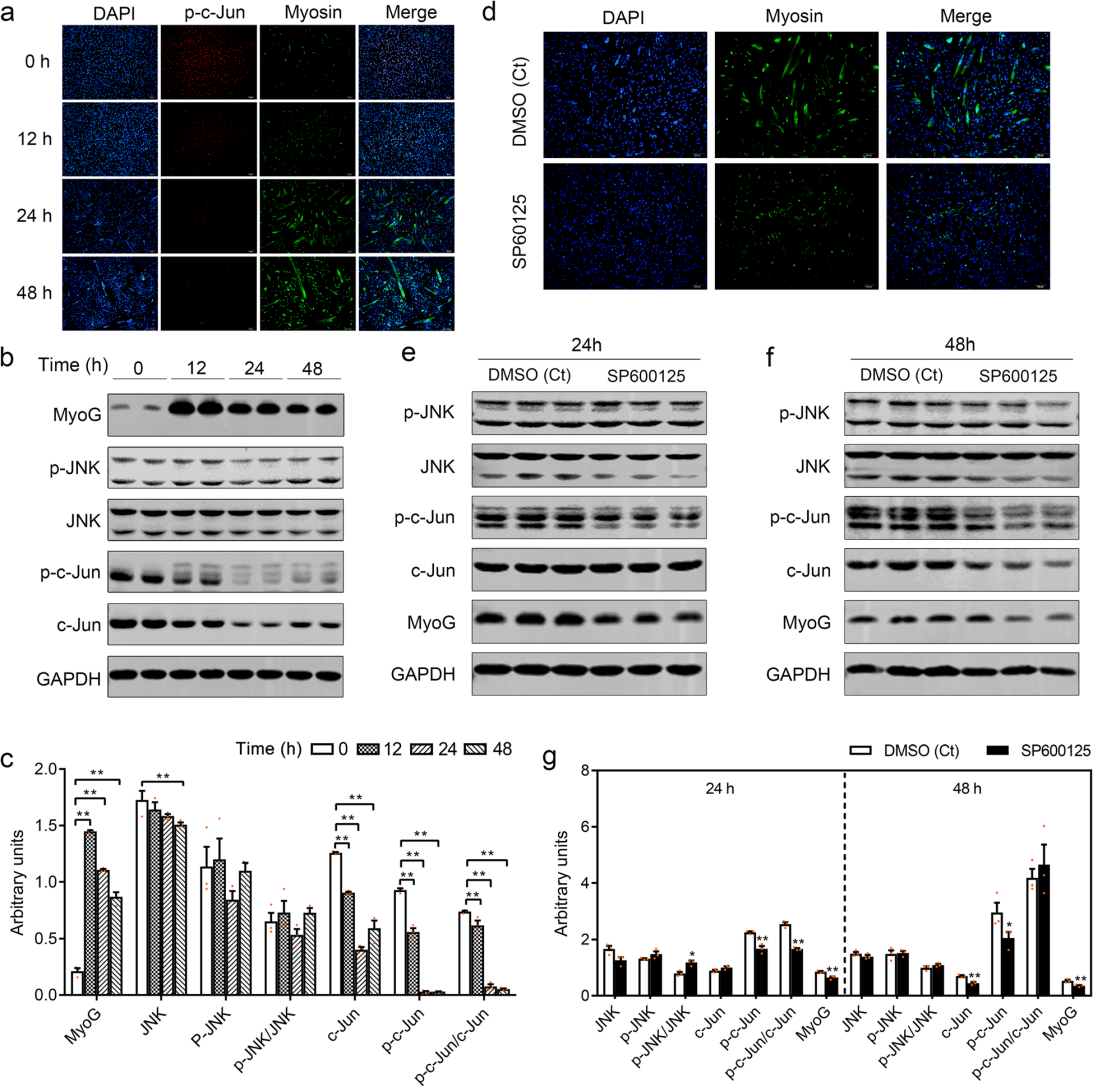
Positive role of JNK/MAPK signaling pathway in regulating myogenic differentiation. **a** Immunofluorescent microscopy analysis of the morphological changes and expression of p-c-Jun and myogenesis marker Myosin in myogenic precursors before and after differentiation. Scale bars, 100 μm. **b** The expression of myogenic marker MyoG, JNK/MAPK signaling key factors (JNK and c-Jun), and their phosphorylated forms were detected by western blotting before and after differentiation. **c** Relative expression level in **b** was calculated. GAPDH was used as the internal control. 0 h represented myogenic precursors in growth medium, and 12, 24, and 48 h represented myogenic precursors switched into differentiation medium for 12, 24, and 48 h, respectively. **d** Representative immunofluorescence images at 48 h after myogenic precursors treated with JNK-specific inhibitor SP600125 or DMSO in differentiation medium. DMSO was used as negative control (Ct). Scale bars, 100 μm. **e**, **f** Expression levels of MyoG, JNK/MAPK signaling key factors (JNK and c-Jun), and their phosphorylated forms (p-JNK and p-c-Jun) were detected by western blotting at 24 and 48 h after cells treated with SP600125 or DMSO in differentiation medium. **g** Relative expression level in **e** and **f** was calculated. GAPDH was used as the internal control. Data were presented as means ± SEM (*n* = 3). The statistical significance of difference between two means was calculated using *t* test, ^*^P < 0.05, ^**^P < 0.01

During myogenic differentiation, along with multinucleated myotubes formation (Fig. 5a) and increased MyoG protein level (Fig. 5b, c), JNK downstream factors p-c-Jun, and total c-Jun drastically decreased demonstrated by immunofluorescence (Fig. 5a) and western blot analysis (Fig. 5b, c), whereas total JNK protein and p-JNK protein were not changed during myogenic differentiation. These results illustrated that JNK/MAPK signaling pathway inactivated during myogenic differentiation.

We further examined the role of JNK/MAPK signaling during myogenic differentiation. After being treated with JNK-specific inhibitor SP600125 at 10 μM in myogenic DM, p-c-Jun/c-Jun significantly decreased at 24 h, while both p-c-Jun and c-Jun significantly decreased at 48 h relative to the control (Fig. 5e–g). Along with the inhibition of JNK/MAPK signaling, myotube formation was significantly suppressed during myogenic induction of myogenic precursors treated with SP600125 and accompanied with a lower expression level of MyoG at 24 and 48 h compared with the control (Fig. 5d–g).

### CACNA2D2-mediated myogenic differentiation through JNK/MAPK signaling pathway

The network linking enriched pathways and hub DEGs outlined the distinct molecular affairs between myogenic and adipogenic precursors (Fig. 4b), in which six co-different genes were involved in MAPK signaling pathway, including calcium voltage-gated channel auxiliary subunit alpha2delta2 (CACNA2D2), filamin C (FLNC), FGF13, FGFR4, PDGFRα, and FGFR2 (Fig. 4b). Notably, mRNA expression levels of CACNA2D2, FLNC, FGF13, and FGFR4 were upregulated in myogenic precursors, while FGFR2 and PDGFRα were downregulated relative to adipogenic precursors. Furthermore, we analyzed the dynamic expression patterns of these six genes during myogenic differentiation. It was noteworthy that mRNA expression levels of PDGFRα, FGFR2, and CACNA2D2 were dramatically decreased (Additional file 2: Figure S2g, h and i), paralleling with the variation of JNK/MAPK signaling pathway during the differentiation of myogenic precursors (Fig. 5a–c).

**Fig. 6:**
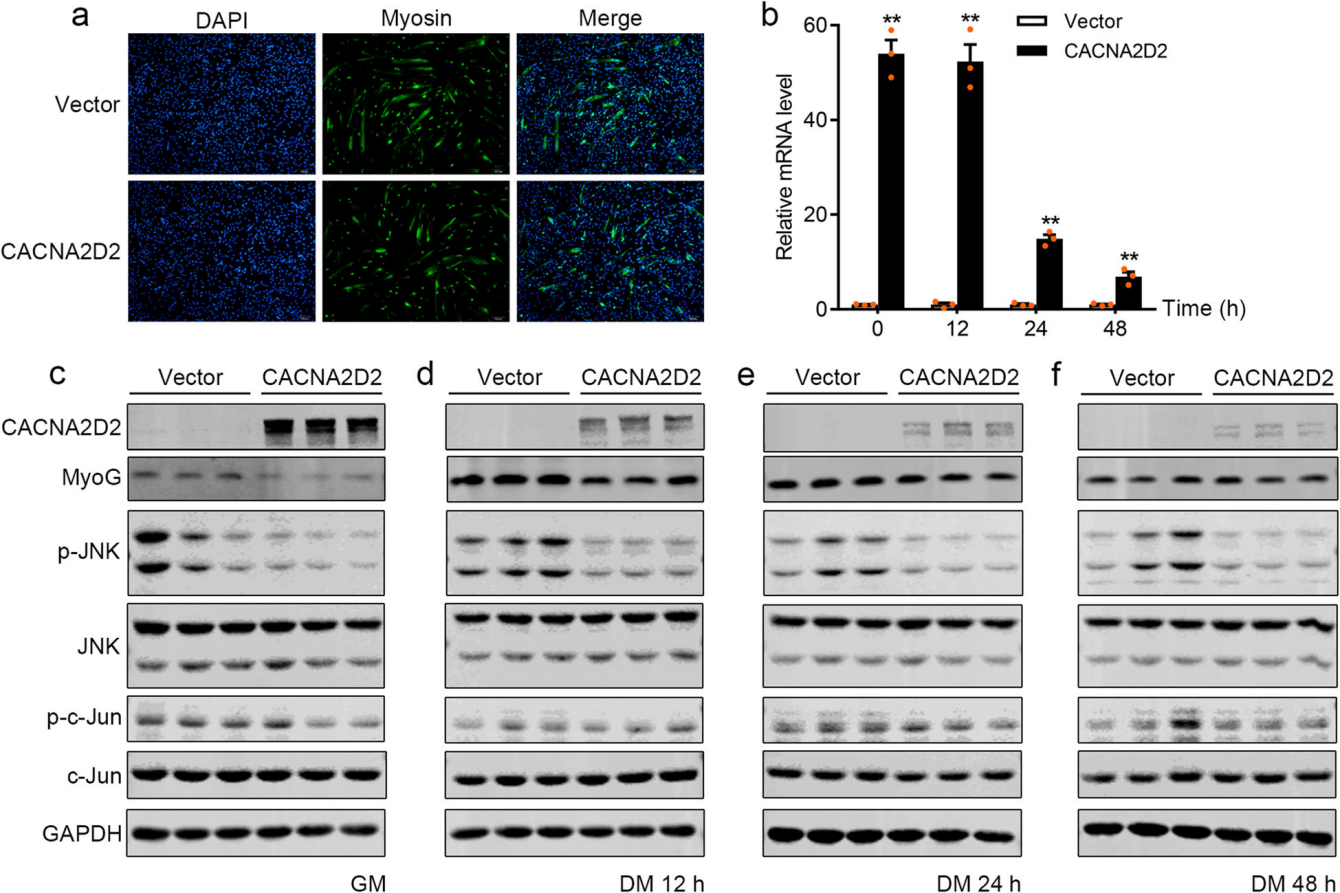
CACNA2D2 negatively regulated myogenic differentiation by inhibiting JNK/MAPK signaling pathway. **a** Immunofluorescent microscopy analysis of the morphological changes and expression of Myosin in myogenic precursors transfected with CACNA2D2-plasmid or empty vector at 48 h of differentiation. Scale bars, 100 μm. **b** The mRNA expression level of *CACNA2D2* and **c**–**f** protein level of CACNA2D2, MyoG, JNK/MAPK signaling key factors (JNK and c-Jun), and their phosphorylated forms (p-JNK and p-c-Jun) were measured in myogenic precursors transfected with CACNA2D2-plasmid or empty vector in growth medium (GM, 0 h) or after 12, 24, and 48 h switched into differentiation medium (DM). GAPDH was used as the internal control. Data were presented as means ± SEM (*n* = 3). The statistical significance of difference between two means was calculated using *t* test, ^**^P < 0.01

Relative to the well-known roles of PDGFRα and FGFR2 in cell differentiation [37, 38], the role of CACNA2D2, especially whether or not regulating myogenic differentiation through JNK/MAPK signaling, remains unknown. Therefore, we explored the function of CACNA2D2 in myogenic differentiation. CACNA2D2 overexpression was successfully achieved during myogenic differentiation, demonstrated by significantly increased CACNA2D2 mRNA and protein expression levels in GM and during the entire period of 48-h myogenic differentiation (Fig. 6b, c). As a result, CACNA2D2-overexpression suppressed multinucleated myotubes formation of myogenic precursors at 48 h of myogenic differentiation (Fig. 6a). Accordingly, MyoG protein level also decreased before or after myogenic differentiation (Fig. 6c, d), suggesting the negative role of CACNA2D2 in myogenic differentiation. Furthermore, overexpression of CACNA2D2 inhibited the phosphorylation of JNK in GM and DM, although the phosphorylation of c-Jun was not changed (Fig. 6c–f). Therefore, CACNA2D2 inhibited myogenic differentiation through the inhibition of JNK/MAPK signaling pathway.

## Discussion

Skeletal muscle is a heterogeneous tissue, consisting of myogenic lineage cells, adipogenic lineage cells, endothelial cells, epithelial cells, immune cells, etc. [39]. As major cell populations, myocytes and adipocytes descend from common progenitors MSCs, and compete during the commitment and determination in the same niche. Impaired skeletal muscle homeostasis in terms of cell populations associated with various myopathies [7, 40, 41]. Excessive adipogenesis and subsequent lipid accumulation in skeletal muscle will undermine muscle functions and metabolism in humans; however, as for farm animals, plenty of lipid accumulating in muscle leads to the improvement of meat quality concerning marbling score. Therefore, the mechanism governing the balance of adipogenesis and myogenesis attracts intensive interest but remains unrevealed yet.

Pigs are a major supplier of animal-derived protein for humans, and pork with high quality embraces high economic value, which affects the consumer’s appetite for fresh pork products. Lean- and obese-type pigs occupy distinct myogenic and adipogenic potential and exhibit different characteristics in lean rate, muscle fiber diameter, and intramuscular fat content [42]. It has been shown that genes concerning fatty acid metabolism and lipid biosynthesis were hyper-methylated in lean-type pigs relative to obese-type pigs, implying DNA methylation implicated in modulation of the balance of myogenesis and adipogenesis in muscle [43]. Besides, pigs are also regarded as a feasible biomedical model and typically applied to explore epigenetic mechanisms of myogenesis and adipogenesis as well as muscle development [44].

During early embryogenesis, to acquire totipotency and create an epigenome for embryonic development, DNA methylation reprogramming occurs, including the erasure of methylation in pre-implantation stages and the subsequent establishment of global DNA methylation upon implantation [45, 46]. It is noting that DNA methylation has critical roles in the maintenance of stem cells identify and differentiation, for example, the differentiation of ESCs is completely inhibited in the absence of DNA methylation [47]. This epigenetic factor can also be decisive in the lineage commitment of MSCs from different origins, including dental pulp and bone marrow [48], and the role of DNA methylation in the lineage commitment of MSCs derived from skeletal muscle still warrants further investigation. Additionally, DNA methylation also promotes activation and differentiation of satellite cells, the adult stem cells in muscle, during early life [49] and restrains its self-renewal during aging [50].

Herein, we presented a comprehensive genome-wide DNA methylation landscape of adipogenic and myogenic precursors derived from skeletal muscle of neonatal pigs to explore the impact of DNA methylation on myogenesis and adipogenesis within skeletal muscle. Due to the lack of antibodies being used in immunofluorescence histochemistry assay for pigs, we confirmed the identification of these two population cells by their different mRNA expression levels of adipocyte-specific genes and myogenic regulatory factors and their distinct differentiation capacity upon adipogenic and myogenic induction (as shown in Figure S1). In addition, in our previous studies, we have evidenced the high purity of these two subpopulation cells [27, 28], and observed significantly higher mRNA expression levels of Desmin and Pax7 in myogenic precursors compared with adipogenic precursors [28]. Based on the significantly divergent differentiation potential, we can reasonably confirm that these two subpopulation cells are adipogenic and myogenic precursors. In this study, 11,361 DMRs, widely distributed in promoter, gene body, and distal intergenic regions, were identified between myogenic and adipogenic precursors. Among them, 52.7% were hypo-methylated, while 47.3% DMRs were hyper-methylated in myogenic precursors relative to adipogenic precursors. The prevalent and distinct distribution of DMRs existing in skeletal muscle global genome implies that DNA methylation plays a critical role in muscle development, which is also supported by the previous studies [17, 51].

To explore the impact of DNA methylation on gene expression, integrated analysis of RRBS and RNA-seq data of the same batch of precursors was performed. DNA methylation is previously described as a silencing signature, but accumulating evidence reveals that DNA methylation may regulate transcription both negatively and positively [12]. In the present study, we identified a total of 153 co-different genes involved in distinct differentiation potential of myogenic and adipogenic precursors and found both negative and positive correlations between mRNA expression and DNA methylation of these co-different genes. For example, DMRs distributed in RyR1 and FGFR2 exhibited the opposite correlation with their mRNA expression level, suggesting the complexity of epigenetic regulation on gene expression existing in skeletal muscle of neonatal pigs.

KEGG analysis based on 153 co-different genes depicted putative regulatory pathways belonging to the cellular process and extracellular environmental processing, through which DNA methylation regulated the distinct differentiation potential of myogenic and adipogenic precursors. These findings were in consistent with our previous study [27]. Among these pathways, focal adhesion pathway, acting as a bridge between integrin-ECM connection and cytoskeleton [52], was demonstrated to be largely affected by DNA methylation status [53]. Besides, pathways belonging to extracellular environmental processing, such as MAPK signaling pathway, Rap1 signaling pathway, PI3K-AKT signaling pathway, and calcium signaling pathway, also contributed to deciphering the discrepancy between myogenic and adipogenic differentiation potential, mirroring the key signaling pathways in regulating skeletal muscle homeostasis. Calcium is an important intracellular signal responsible for mediating various biological events, including adipogenesis [54], myogenesis [55], and the development of muscular dystrophy [56]. Consistent with our previous studies [28, 57], calcium signaling pathway was also enriched with co-different genes, suggesting a close interaction between calcium signaling pathway and the balance of myogenesis and adipogenesis in skeletal muscle. In particular, we observed distinct patterns of RyR1 and ATP2B3 in terms of DNA methylation and gene expression between myogenic and adipogenic precursors. Although hyper-methylation-induced RyR1 silencing was unveiled in skeletal muscle of patients with recessive core myopathies [58], our knowledge concerning the epigenetic regulation in RyR1-mediated adipogenesis and myogenesis within skeletal muscle remains limited, let alone ATP2B3.

Considering the high number of co-different genes involved in MAPK signaling pathway, we paid more attention to impacts of MAPK signaling pathway on cell fate commitment in the present study and observed significantly enhanced JNK/MAPK signaling in myogenic precursors compared with adipogenic precursors. JNK/MAPK inhibition by SP600125 further indicated a positive role of JNK/MAPK signaling in myogenic differentiation, which was in concert with the role of JNK/MAPK signaling in stimulating myofibers growth through myostatin/SMAD inhibition [24]. On the contrary, a negative role of JNK/MAPK signaling in myogenic differentiation was also observed in mouse C2C12 myoblast cells [29]. A recent study performed in chickens demonstrated that intramuscular adipogenic precursors might impede differentiation of satellite cells via JNK/MAPK pathway [59], further encouraging studies of the complicated roles of JNK/MAPK pathway in the crosstalk of myogenic and adipogenic precursors within skeletal muscle. Notably, among six co-different genes (FGF13, FLNC, PDGFRα, FGFR2, FGFR4, and CACNA2D2) involved in JNK/MAPK signaling pathway, the dynamic expression patterns of PDGFRα, FGFR2, and CACNA2D2 paralleled to the alteration of JNK/MAPK pathway during myogenic differentiation. Studies have evidenced the positive role of FLNC in the differentiation and fusion of C2C12 myoblasts and myofiber development of mice [60]. Similar to the function of FLNC, FGFR2, and FGFR4 were reported to support skeletal muscle development [38], myogenic differentiation [61], and muscle regeneration [62]. In contrast, FGF13 inhibited myogenic differentiation of C2C12 myoblasts by activating ERK1/2 signaling [63]. Different from functions of the above genes in myogenesis, PDGFRα^+^ mesenchymal progenitors were the major contributor to ectopic fat formation in skeletal muscle [37], and embryonic adipose progenitor cells without PDGFRα would undergo fate change from adipogenic to fibrotic lineage [64]. Although DNA methylation-induced PDGFRα and FGFR2 silencing has been reported in CG4 cells [65] or primary human pituitary adenomas [66], the role of DNA methylation in regulating the expression level of these co-different genes and their effects on myogenesis or adipogenesis still remains unclear. Unlike the well-known functions of the above genes in ectopic fat accumulation and skeletal muscle development, the function of CNCNA2D2 in relation to skeletal muscle development and homeostasis has been sparsely described in previous studies. Our study revealed that CACNA2D2 suppressed myogenic differentiation and myotube formation through inhibiting the phosphorylation of JNK/MAPK, while the phosphorylation of c-Jun was not altered by CACNA2D2 overexpression. Of note, JNK/MAPK could directly phosphorylate nuclear factor of activated T-cell (NFAT) [67, 68], transcription factors regulated by intracellular Ca^2+^ signaling [69]. Interestingly, CACNA2D2-induced increase of cytosolic-free Ca^2+^ content was observed in cancer cells [70, 71]. Therefore, CACNA2D2 probably mediates NFAT by inhibiting JNK/MAPK signaling during myogenic differentiation. Therefore, the mechanism of CACNA2D2-suppressed myogenic differentiation warrants future studies. In one word, we demonstrated the new function of CACNA2D2 that impeded myogenic differentiation through the inhibition of JNK/MAPK signaling pathway in the present study, but the epigenetic regulation of CACNA2D2 during cell fate commitment and differentiation still merits further clarification.

## Conclusions

This study provided a comprehensive landmark of DNA methylome of skeletal muscle-derived myogenic and adipogenic precursors and highlighted that DNA methylation influenced pathways belonging to cellular process and extracellular environmental processing, thereby mediating cell fate commitment and skeletal muscle homeostasis. Particularly, we found that CACNA2D2 might be involved in the regulation of myogenic differentiation via suppressing JNK/MAPK signaling in a DNA methylation-dependent manner. This work will help uncover the molecular mechanism that mediates the balance of myogenesis and adipogenesis in skeletal muscle of pigs, and promote the further application of pig as a model organism for research concerning skeletal muscle diseases in humans.

## Supplementary Information

**Additional file 1: Figure S1.** Isolation and identification of adipogenic and myogenic precursors from porcine skeletal muscle by preplate technique. (a) Morphology in growth medium, (b) immunofluorescence and (c) Oil Red O staining of adipogenic and myogenic precursors following 2 d of myogenic differentiation and 9 d of adipogenic differentiation, respectively. Myosin (green), DAPI (blue), and Oil Red O (red). Scale bars for a and c, 200 μm. Scale bars for b, 100 μm. (d) Gene expression analysis (*n* = 3) of myogenic and adipogenic commitment related genes by RNA-seq. (e) Quantitative analysis of lipid droplet by optical density of Oil Red O after adipogenic differentiation on d 9. Data were presented as means ± SEM (*n* = 3). The statistical significance of difference between two means was calculated using *t*-test, ^**^P < 0.01. Adi, adipogenic precursors. Myo, myogenic precursors.

**Additional file 2: Figure S2.** Expression patterns of myogenic markers and co-different genes enriched in MAPK pathway during myogenic differentiation. The mRNA expression level of myogenic markers, including (a-d) *Myf5*, *MyoD1*, *MyoG*, and *Myomarker*, and six co-different genes enriched in MAPK signaling pathway, such as (e-j) *FGF13*, *FLNC*, *PAGFRA*, *FGFR2*, *FGFR4*, and *CACNA2D2*, were determined by qRT-PCR assays before differentiation (0 h) or at 12, 24, and 48 h after differentiation. *GAPDH* was used as the internal control. Results were presented as means ± SEM (*n* = 3). Different letters between bars mean *P* < 0.05 in one-way ANOVA analyses followed by post hoc Tukey’s tests.

**Additional file 3: Table S1.** Primer sequences used for qRT-PCR analysis.

**Additional file 4: Table S2.** Quality control of reduced representation bisulfite sequencing (RRBS).

**Additional file 5: Table S3.** 153 Genes with different DNA methylation and mRNA expression levels between myogenic and adipogenic precursors.

**Additional file 6: Table S4.** Transcription factors (TFs) with different expression level between adipogenic and myogenic precursors.

**Additional file 7: Table S5.** Transcription factors (TFs) enriched with differentially methylated regions (DMRs) between adipogenic and myogenic precursors.

## Data Availability

All data generated or analyzed during this study are included in this published article and its supplementary information files. The datasets used and analyzed during the current study are available from the corresponding author on reasonable request.

## Abbreviations

BSA: Bovine serum albumin
C/EBPα: CCAAT/enhancer binding protein alpha
CACNA2D2: Calcium voltage-gated channel auxiliary subunit alpha2delta2
CGIs: CpG islands
DEGs: Differentially expressed genes
DM: Differentiation medium
DMRs: Differentially methylated regions
DNMT1: DNA methyltransferase 1
ERK: Extracellular signal-related kinases
ESCs: Embryonic stem cells
FGF13: Fibroblast growth factor 13
FGFR: Fibroblast growth factor receptor
FLNC: Filamin C
GM: Growth medium
ITGA9: Integrin subunit alpha 9
JNK: c-Jun amino-terminal kinases
KEGG: Kyoto Encyclopedia of Genes and Genomes
MAPK: Mitogen-activated protein kinase
MEF2C: Myocyte enhancer factor 2C
MET: MET proto-oncogene, receptor tyrosine kinase
MSCs: Mesenchymal stem cells
Myf5: Myogenic factor 5
MyoD1: Myoblast determination protein 1
MyoG: Myogenin
NFAT: Nuclear factor of activated T-cell
OD: Optical density
PDGFRα: Platelet-derived growth factor receptor alpha
PPARγ: Peroxisome proliferator-activated receptor gamma
PVDF: Polyvinylidene difluoride
RRBS: Reduced representation bisulfite sequencing
RT: Room temperature
TFs: Transcription factors
TSS: Transcription start site
