# Supplementary figures and images for: Global DNA methylation pattern involved in the modulation of differentiation potential of adipogenic and myogenic precursors in skeletal muscle of pigs
Source: Stem Cell Res Ther. 2020 Dec 11;11:536. doi: 10.1186/s13287-020-02053-3 (PMC7731745; doi:10.1186/s13287-020-02053-3)

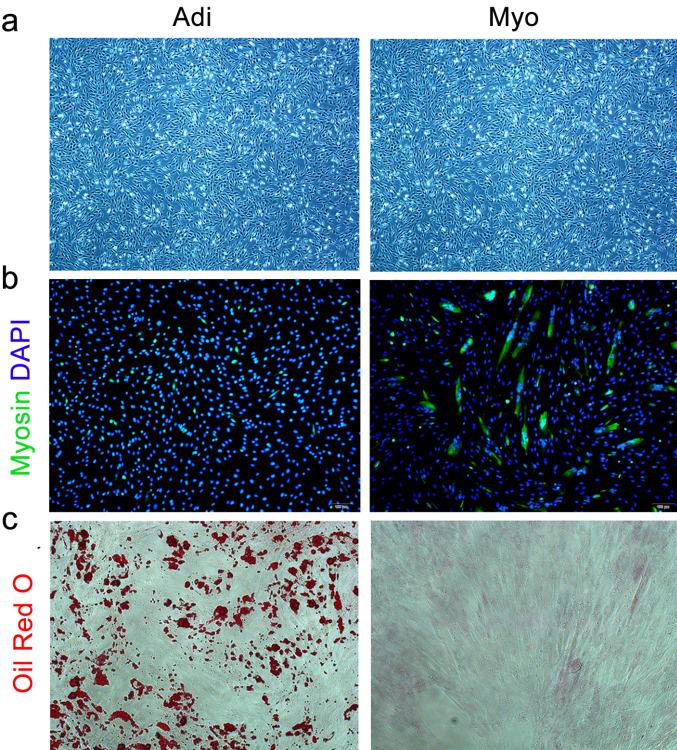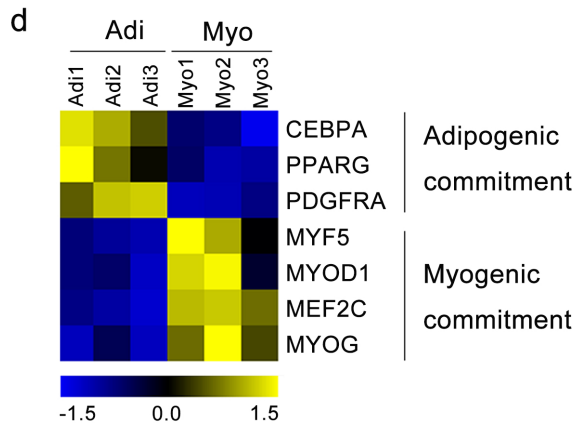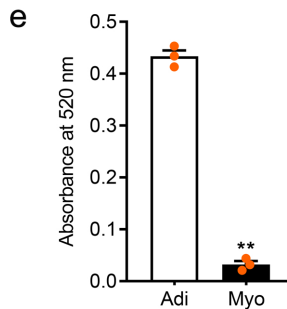

Supplement: Supplementary file 1 — Additional file 1: Figure S1. Isolation and identification of adipogenic and myogenic precursors from porcine skeletal muscle by preplate technique. (a) Morphology in growth medium, (b) immunofluorescence and (c) Oil Red O staining of adipogenic and myogenic precursors following 2 d of myogenic differentiation and 9 d of adipogenic differentiation, respectively. Myosin (green), DAPI (blue), and Oil Red O (red). Scale bars for a and c, 200 μm. Scale bars for b, 100 μm. (d) Gene expression analysis (n = 3) of myogenic and adipogenic commitment related genes by RNA-seq. (e) Quantitative analysis of lipid droplet by optical density of Oil Red O after adipogenic differentiation on d 9. Data were presented as means ± SEM (n = 3). The statistical significance of difference between two means was calculated using t-test, **P < 0.01. Adi, adipogenic precursors. Myo, myogenic precursors. [file 13287_2020_2053_MOESM1_ESM.pdf]

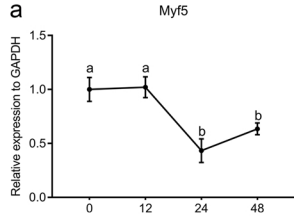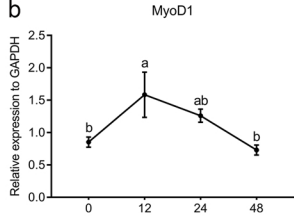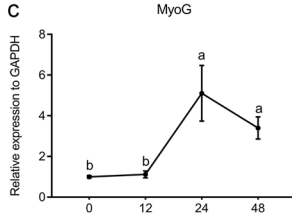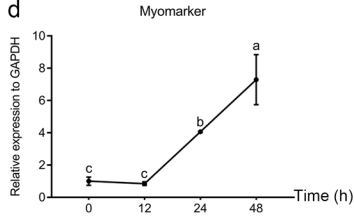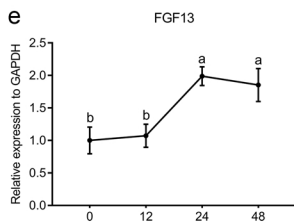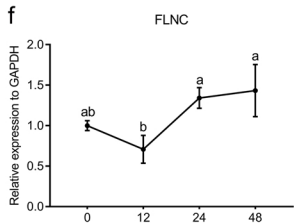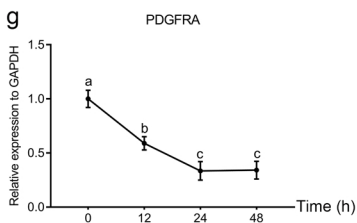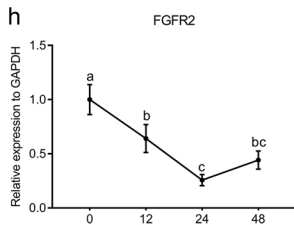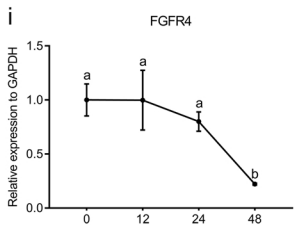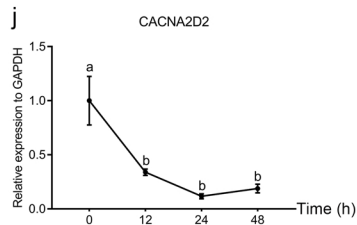

Supplement: Supplementary file 2 — Additional file 2: Figure S2. Expression patterns of myogenic markers and co-different genes enriched in MAPK pathway during myogenic differentiation. The mRNA expression level of myogenic markers, including (a-d) Myf5, MyoD1, MyoG, and Myomarker, and six co-different genes enriched in MAPK signaling pathway, such as (e-j) FGF13, FLNC, PAGFRA, FGFR2, FGFR4, and CACNA2D2, were determined by qRT-PCR assays before differentiation (0 h) or at 12, 24, and 48 h after differentiation. GAPDH was used as the internal control. Results were presented as means ± SEM (n = 3). Different letters between bars mean P < 0.05 in one-way ANOVA analyses followed by post hoc Tukey’s tests. [file 13287_2020_2053_MOESM2_ESM.pdf]
