# Supplementary material for: Global DNA methylation pattern involved in the modulation of differentiation potential of adipogenic and myogenic precursors in skeletal muscle of pigs
Source: Stem Cell Res Ther. 2020 Dec 11;11:536. doi: 10.1186/s13287-020-02053-3 (PMC7731745; doi:10.1186/s13287-020-02053-3)
Supplement: Supplementary file 3 — Additional file 3: Table S1. Primer sequences used for qRT-PCR analysis. [file 13287_2020_2053_MOESM3_ESM.pdf]

Table S1. Primer sequences used for qRT-PCR analysis.

Gene	Primer sequence, 5'-3'		Product size (bp)
	Forward	Reverse	
Myf5	AGTTCGGGGACGAGTTTGAG	TCAAACGCCTGGTTGACCTT	232
MyoD1	GGTGACTCAGACGCATCCAG	AGGTGCCGTCGTAGCAGTTC	106
Myogenin	CTGCTCACAGCTGACCCTAC	GGTTTCATCTGGGAAGGCCA	105
Myomarker	CAGCAGATGAAGGACCAACG	CGCAGTGGTAGAAGCTGTGG	154
FGF13	GCCTTCGAGTGGTGGCTATTCAG	CCAGACTGCTGCTGACGGTATATC	175
FLNC	TCCAGGAACCAGGTGACTATGAGG	CAGTAAGACGGCGAGCATCATCC	114
PDGFRα	ATCGTGGAGAATCTGCTGCCTG	GATGATGTAGCCGCTGTCTG	216
FGFR2	TCCATCGAGATTTAGCCGCC	TGGGTGTACACGCGATCAAA	178
FGFR4	CTCACAGTGTTGCCAGAAGAGGAC	AGCCAGCAGCAGAAGCACAAG	120
CACNA2D2	CGCCTCCTACAACGCCATCATC	AAGAGAAGGTTGGTGTTGGTCAGC	84
GAPDH	TCGGAGTGAACGGATTTG	CCTGGAAGATGGTGATGG	219
