# Supplementary material for: Global DNA methylation pattern involved in the modulation of differentiation potential of adipogenic and myogenic precursors in skeletal muscle of pigs
Source: Stem Cell Res Ther. 2020 Dec 11;11:536. doi: 10.1186/s13287-020-02053-3 (PMC7731745; doi:10.1186/s13287-020-02053-3)
Supplement: Supplementary file 4 — Additional file 4: Table S2. Quality control of reduced representation bisulfite sequencing (RRBS). [file 13287_2020_2053_MOESM4_ESM.pdf]

Table S2. Quality control of reduced representation bisulfite sequencing (RRBS).

Sample	Clean-Reads	Q20 (%)	Unique-mapped	Mapped (%)	Conversion rate (%)
Adi1	15,028,826	96.84	7,471,019	49.71	99.63
Adi2	13,904,012	96.72	7,083,110	50.94	99.67
Adi3	13,653,176	96.79	7,026,427	51.46	99.56
Myo1	13,418,196	97.48	6,693,519	49.63	99.57
Myo2	13,747,164	97.5	6,853,831	49.86	99.59
Myo3	14,109,859	97.51	7,372,166	52.25	99.59
