# Supplementary material for: Global DNA methylation pattern involved in the modulation of differentiation potential of adipogenic and myogenic precursors in skeletal muscle of pigs
Source: Stem Cell Res Ther. 2020 Dec 11;11:536. doi: 10.1186/s13287-020-02053-3 (PMC7731745; doi:10.1186/s13287-020-02053-3)
Supplement: Supplementary file 5 — Additional file 5: Table S3. 153 Genes with different DNA methylation and mRNA expression levels between myogenic and adipogenic precursors. [file 13287_2020_2053_MOESM5_ESM.pdf]

Table S3. 153 Genes with different DNA methylation and mRNA expression levels between myogenic and adipogenic precursors.

Ensembl Gene ID	Gene Name	Description
ENSSSCG00000000029	SCUBE1	Signal peptide, CUB domain, EGF-like 1
ENSSSCG00000000033	TSPO	Translocator protein
ENSSSCG00000000162	BTBD11	BTB (POZ) domain containing 11
ENSSSCG00000000735	TSPAN9	Tetraspanin 9
ENSSSCG00000000937	MYF5	Myogenic factor 5
ENSSSCG00000001004	SLC22A23	Solute carrier family 22, member 23
ENSSSCG00000001050	EDN1	Endothelin 1
ENSSSCG00000001052	PHACTR1	Phosphatase and actin regulator 1
ENSSSCG00000001066	RBM24	RNA binding motif protein 24
ENSSSCG00000001597	LRFN2	Leucine rich repeat and fibronectin type III domain containing 2
ENSSSCG00000001751	CHRNA5	Cholinergic receptor, nicotinic, alpha 5
ENSSSCG00000001787	IL6	Interleukin 16
ENSSSCG00000002555	JAG2	Jagged 2
ENSSSCG00000002557	NUDT14	Nudix hydrolase 14
ENSSSCG00000002637	DBNDD1	Dysbindin (dystrobrevin binding protein 1) domain containing 1
ENSSSCG00000002826	-	Uncharacterized protein
ENSSSCG00000002831	IRX3	Iroquois homeobox 3
ENSSSCG00000002836	SALL1	Spalt-like transcription factor 1
ENSSSCG00000002961	RYR1	Ryanodine receptor 1 (skeletal)
ENSSSCG00000003022	TMEM145	Transmembrane protein 145
ENSSSCG00000003061	CADM4	Cell adhesion molecule 4
ENSSSCG00000003143	MAMSTR	MEF2 activating motif and SAP domain containing transcriptional regulator
ENSSSCG00000003437	TNFRSF8	Tumor necrosis factor receptor superfamily, member 8
ENSSSCG00000003470	FAM131C	Family with sequence similarity 131, member C
ENSSSCG00000003595	SERINC2	Serine incorporator 2
ENSSSCG00000003693	MYOM1	Myomesin 1
ENSSSCG00000004273	COL19A1	Collagen, type XIX, alpha 1
ENSSSCG00000004479	FILIP1	Filamin A interacting protein 1
ENSSSCG00000004713	TGM5	Transglutaminase 5
ENSSSCG00000005240	DOCK8	Dedicator of cytokinesis 8
ENSSSCG00000005278	PRUNE2	Prune homolog 2 (Drosophila)
ENSSSCG00000005358	SHB	SH2 domain containing adaptor protein B
ENSSSCG00000005375	CORO2A	Coronin, actin binding protein 2A
ENSSSCG00000005480	-	Uncharacterized protein
ENSSSCG00000005593	OLFML2A	Olfactomedin like 2A

ENSSSCG00000005627	AK1	Adenylate kinase isoenzyme 1
ENSSSCG00000006059	NCALD	Neurocalcin delta
ENSSSCG00000006156	TPD52	Tumor protein D52
ENSSSCG00000006542	KCNN3	Potassium channel, calcium activated intermediate/small conductance subfamily N alpha, member 3
ENSSSCG00000006874	PALMD	Palmdelphin
ENSSSCG00000006893	BCAR3	Breast cancer anti-estrogen resistance 3
ENSSSCG00000006911	TGFBR3	Transforming growth factor beta receptor type 3 precursor
ENSSSCG00000007200	FAM110A	Family with sequence similarity 110, member A
ENSSSCG00000007470	FAM65C	Family with sequence similarity 65, member C
ENSSSCG00000007482	TSHZ2	Teashirt zinc finger homeobox 2
ENSSSCG00000007485	BCAS1	Breast carcinoma amplified sequence 1
ENSSSCG00000007509	PMEPA1	Prostate transmembrane protein, androgen induced 1
ENSSSCG00000008446	SIX2	SIX homeobox 2
ENSSSCG00000008702	-	Uncharacterized protein
ENSSSCG00000008984	SHROOM3	Shroom family member 3
ENSSSCG00000009131	PITX2	Paired-like homeodomain 2
ENSSSCG00000009229	ARHGAP24	Rho GTPase activating protein 24
ENSSSCG00000009320	FLT1	Fms-related tyrosine kinase 1
ENSSSCG00000009331	MEDAG	Mesenteric estrogen-dependent adipogenesis
ENSSSCG00000009535	EFNB2	Ephrin-B2
ENSSSCG00000009603	LZTS1	Leucine zipper, putative tumor suppressor 1
ENSSSCG00000009655	EBF2	Early B-cell factor 2
ENSSSCG00000010144	ACTN2	Actin alpha 2
ENSSSCG00000010199	RET	Ret proto-oncogene
ENSSSCG00000010212	ANK3	Ankyrin 3, node of Ranvier
ENSSSCG00000010272	ADAMTS14	ADAM metallopeptidase with thrombospondin type 1 motif 14
ENSSSCG00000010359	LDB3	LIM domain binding 3
ENSSSCG00000010376	GDF10	Growth differentiation factor 10
ENSSSCG00000010447	ACTA2	Actin alpha 2
ENSSSCG00000010578	PITX3	Paired-like homeodomain 3
ENSSSCG00000010698	FGFR2	Fibroblast growth factor receptor 2
ENSSSCG00000010749	FAM196A	Family with sequence similarity 196, member A
ENSSSCG00000010755	PTPRE	Protein tyrosine phosphatase, receptor type, E
ENSSSCG00000010943	-	Uncharacterized protein
ENSSSCG00000010974	CNTFR	Ciliary neurotrophic factor receptor
ENSSSCG00000011259	SCN5A	Sodium channel, voltage gated, type V alpha subunit
ENSSSCG00000011412	CACNA2D2	Calcium voltage-gated channel auxiliary subunit alpha2delta2
ENSSSCG00000011463	IL17RD	Interleukin 17 receptor D
ENSSSCG00000011579	PPARγ	Peroxisome proliferator-activated receptor gamma
ENSSSCG00000011646	KY	Kyphoscoliosis peptidase

ENSSSCG00000011934	PLCXD2	Phosphatidylinositol-specific phospholipase C, X domain containing 2
ENSSSCG00000012083	RIPK4	Receptor-interacting serine-threonine kinase 4
ENSSSCG00000012110	MID1	Midline 1
ENSSSCG00000012238	TSPAN7	Tetraspanin 7
ENSSSCG00000012607	TMEM255A	Transmembrane protein 255A
ENSSSCG00000012644	TENM1	Teneurin transmembrane protein 1
ENSSSCG00000012680	GPC3	Glypican 3
ENSSSCG00000012769	ATP2B3	ATPase, Ca^{2+} transporting, plasma membrane 3
ENSSSCG00000012869	LOC100518411	Anoctamin
ENSSSCG00000013113	TMEM132A	Transmembrane protein 132A
ENSSSCG00000013341	LUZP2	Leucine zipper protein 2
ENSSSCG00000013354	CSRP3	Cysteine and glycine-rich protein 3
ENSSSCG00000013375	MYOD1	Myogenic differentiation 1
ENSSSCG00000014012	GFPT2	Glutamine-fructose-6-phosphate transaminase 2
ENSSSCG00000014047	FGFR4	Fibroblast growth factor receptor 4
ENSSSCG00000014242	ZNF608	Zinc finger protein 608
ENSSSCG00000014251	MEGF10	Multiple EGF-like-domains 10
ENSSSCG00000014316	TGFBI	Transforming growth factor, beta-induced
ENSSSCG00000014430	ABLIM3	Actin binding LIM protein family, member 3
ENSSSCG00000014575	SCUBE2	Signal peptide, CUB domain, EGF-like 2
ENSSSCG00000014920	FZD4	Frizzled class receptor 4
ENSSSCG00000015116	MCAM	Melanoma cell adhesion molecule
ENSSSCG00000015249	ADAMTS8	ADAM metallopeptidase with thrombospondin type 1 motif, 8
ENSSSCG00000015475	MYOG	Myogenin
ENSSSCG00000015706	LYPD1	LY6/PLAUR domain containing 1
ENSSSCG00000015774	TENM3	Teneurin transmembrane protein 3
ENSSSCG00000015802	FAM149A	Family with sequence similarity 149, member A
ENSSSCG00000016002	CCDC141	Coiled-coil domain containing 141
ENSSSCG00000016288	CHRNG	Cholinergic receptor, nicotinic, gamma
ENSSSCG00000016381	SNED1	Sushi, nidogen and EGF-like domains 1
ENSSSCG00000016578	FLNC	Filamin C
ENSSSCG00000016633	MET	MET proto-oncogene, receptor tyrosine kinase
ENSSSCG00000016672	ADCYAP1R1	Adenylate cyclase activating polypeptide 1 (pituitary) receptor type I
ENSSSCG00000016720	PGAM2	Phosphoglycerate mutase 2
ENSSSCG00000017012	SLIT3	Slit guidance ligand 3
ENSSSCG00000017052	ADAM19	ADAM metallopeptidase domain 19
ENSSSCG00000017054	CYFIP2	Cytoplasmic FMR1 interacting protein 2
ENSSSCG00000017095	SEMA5A	Sema domain, seven thrombospondin repeats (type 1 and type 1-like), transmembrane domain (TM) and short cytoplasmic domain

		(semaphorin) 5A
ENSSSCG00000017141	AATK	Apoptosis-associated tyrosine kinase
ENSSSCG00000017164	TIMP2	TIMP metallopeptidase inhibitor 2
ENSSSCG00000017181	CYGB	Cytoglobin
ENSSSCG00000017299	MARCH10	Membrane-associated ring finger (C3HC4) 10, E3 ubiquitin protein ligase
ENSSSCG00000017585	SAMD14	Sterile alpha motif domain containing 14
ENSSSCG00000017783	-	-
ENSSSCG00000017874	ATP2A3	ATPase sarcoplasmic/endoplasmic reticulum Ca^{2+} transporting 3
ENSSSCG00000017894	WSCD1	WSC domain containing 1
ENSSSCG00000017904	ENO3	Enolase 3
ENSSSCG00000021111	LOC100525453	Dihydropyrimidinase-related protein 3
ENSSSCG00000021375	LOC100627069	Heparan sulfate 6-O-sulfotransferase 2
ENSSSCG00000021576	CD83	CD83 molecule
ENSSSCG00000021606	FLVCR2	Feline leukemia virus subgroup C cellular receptor family member 2
ENSSSCG00000021624	LAD1	Ladinin 1
ENSSSCG00000021899	PIEZO2	Piezo-type mechanosensitive ion channel component 2
ENSSSCG00000022280	DACT3	Dishevelled-binding antagonist of beta-catenin 3
ENSSSCG00000022331	FGF13	Fibroblast growth factor 13
ENSSSCG00000022592	FIBIN	Fin bud initiation factor homolog (zebrafish)
ENSSSCG00000022850	LOC100522032	Rhomboid-related protein 3
ENSSSCG00000023611	TNXB	Tenascin XB
ENSSSCG00000024045	GATSL2	GATS protein like 2
ENSSSCG00000024528	ZDHHC14	Zinc finger, DHHC-type containing 14
ENSSSCG00000024651	HEYL	Hes-related family bHLH transcription factor with YRPW motif-like
ENSSSCG00000024669	NBL1	DAN family BMP antagonist
ENSSSCG00000024676	SRPK3	SRSF protein kinase 3
ENSSSCG00000024719	-	Uncharacterized protein
ENSSSCG00000025353	TNNT1	Troponin T type 1 (skeletal, slow)
ENSSSCG00000025924	IGFBP5	Uncharacterized protein
ENSSSCG00000026602	PTGIR	Prostaglandin I2 (prostacyclin) receptor
ENSSSCG00000026732	-	Uncharacterized protein
ENSSSCG00000026868	LRRC15	Leucine rich repeat containing 15
ENSSSCG00000027204	LOC102164256	Collagen alpha-1(XV) chain-like
ENSSSCG00000027365	WNT7B	Wingless-type MMTV integration site family, member 7B
ENSSSCG00000027935	FHOD3	Formin homology 2 domain containing 3
ENSSSCG00000028873	PDGFRα	Platelet-derived growth factor receptor alpha
ENSSSCG00000029234	ZNF536	Zinc finger protein 536
ENSSSCG00000029890	RAPSN	Receptor-associated protein of the synapse

ENSSSCG00000030110	-	Uncharacterized protein
ENSSSCG00000030516	ITGA9	Integrin alpha 9
ENSSSCG00000030597	HAPLN3	Hyaluronan and proteoglycan link protein 3
