# Supplementary material for: Global DNA methylation pattern involved in the modulation of differentiation potential of adipogenic and myogenic precursors in skeletal muscle of pigs
Source: Stem Cell Res Ther. 2020 Dec 11;11:536. doi: 10.1186/s13287-020-02053-3 (PMC7731745; doi:10.1186/s13287-020-02053-3)
Supplement: Supplementary file 6 — Additional file 6: Table S4. Transcription factors (TFs) with different expression level between adipogenic and myogenic precursors. [file 13287_2020_2053_MOESM6_ESM.pdf]

Table S4. Transcription factors (TFs) with different expression level between adipogenic and myogenic precursors.

Ensembl Gene ID	Gene name	TFs family	Fold change (Myo/Adi)	Lineages
ENSSSCG00000008123	ARID5A	ARID	2.25	Chondrocyte
ENSSSCG00000000937	MYF5	bHLH	3.66	Myocyte
ENSSSCG00000006159	HEY1	bHLH	2.81	Multiple
ENSSSCG00000006222	BHLHE2	bHLH	2.79	Thymocyte
ENSSSCG00000009703	HAND2	bHLH	4.44	Cardiomyocyte
ENSSSCG00000013375	MYOD1	bHLH	2.36	Myocyte
ENSSSCG00000015475	MYOG	bHLH	2.75	Myocyte
ENSSSCG00000002866	CEBPα	C/EBP	0.40	Adipocyte
ENSSSCG00000009655	EBF2	COE	0.30	Adipocyte
ENSSSCG00000000062	CSDC2	CSD	2.25	Neuron
ENSSSCG00000006235	TOX	HMG	4.50	-
ENSSSCG00000008649	SOX11	HMG	3.10	Neuron
ENSSSCG00000002831	IRX3	Homeobox	2.19	Neuron
ENSSSCG00000005087	SIX1	Homeobox	2.01	Myocyte
ENSSSCG00000008446	SIX2	Homeobox	3.41	Myocyte
ENSSSCG00000010578	PITX3	Homeobox	4.20	Neuron
ENSSSCG00000011579	PPARγ	PPAR	0.39	Adipocyte
ENSSSCG00000014149	MEF2C	SRF	2.48	Myocyte
ENSSSCG00000011211	THRB	Thyroid	2.99	-
ENSSSCG00000011936	ZBED2	zf-BED	2.38	Myocyte
ENSSSCG00000002836	SALL1	zf-C2H2	0.46	Neuron
ENSSSCG00000002838	ZNF423	zf-C2H2	0.50	Adipocyte
ENSSSCG00000009462	KLF5	zf-C2H2	2.41	Adipocyte
