# Supplementary material for: Global DNA methylation pattern involved in the modulation of differentiation potential of adipogenic and myogenic precursors in skeletal muscle of pigs
Source: Stem Cell Res Ther. 2020 Dec 11;11:536. doi: 10.1186/s13287-020-02053-3 (PMC7731745; doi:10.1186/s13287-020-02053-3)
Supplement: Supplementary file 7 — Additional file 7: Table S5. Transcription factors (TFs) enriched with differentially methylated regions (DMRs) between adipogenic and myogenic precursors. [file 13287_2020_2053_MOESM7_ESM.pdf]

Table S5. Transcription factors (TFs) enriched with differentially methylated regions (DMRs) between adipogenic and myogenic precursors.

DMRs terms	Motifs	TFs that binding to motifs
**Hypo promoter**	Motif 2	ZNF263
	Motif 6	Foxk1
	Motif 7	ZNF740, SP1/3, RREB1, TFAP2A, E2F4, ZNF263, KLF4/5, EGR1
**Hyper promoter**	Motif1	SVP, ID1, Foxk1
	Motif 2	SVP, Foxk1, IRF1
	Motif 3	ZNF740, SP1/3, RREB1, E2F4, KLF4/5, PLAG1, ZNF263, PPARγ, EGR1, RARA
	Motif 4	ZNF740, SP1, RREB1, KLF4, ZNF263, KLF5, PPARγ, EGR1, SP3
	Motif 7	RELA
	Motif 14	SVP
**Hypo 3'-UTR**	Motif 1	SVP, ID1, Foxk1, IRF1
	Motif 2	LEF1, SVP, Foxp2
	Motif 3	SP1, ZNF263, PPARγ, EGR1
	Motif 4	ZNF740, SP1, E2F4, ABF1, ZNF263, KLF5, PPARγ, EGR1
**Hyper 3'-UTR**	Motif 1	SVP, Foxk1
	Motif 2	ZNF740, SP1, E2F4, ZNF263, EGR1
	Motif 3	SP1, RXRB, C/EBPα, KLF4, KLF5, PPARγ, NR2C2, Nr2f6, SP4, SP3
	Motif 4	Foxk1, SVP
	Motif 7	ZNF740, SP1, RREB1, E2F4, ZNF263
	Motif 8	FKH
	Motif 9	FOXP2, Hoxa11
**Hypo 5'-UTR**	Motif 1	PLAG1
	Motif 3	USF1/2, TCF21, ABF1, MSC, ARNT, ID2, TCF3/4, MYOG, CREB3L2
	Motif 11	MEIS3, PBX1
**Hyper 5'-UTR**	Motif 2	E2F4, ZNF263, EGR1
	Motif 4	FOXP2
	Motif 6	SPI1
	Motif 10	Foxo1
	Motif 15	NFAT5, NFATC1, NFATC2, ID1
**Hypo exon**	Motif 2	SVP, Foxk1
	Motif 3	SVP
	Motif 4	ZNF740, SP1, ZNF263, KLF5, EGR1
**Hyper exon**	Motif 1	SP1, ZNF263, MYOD1, MYOG
	Motif 2	SP1, E2F4, ZNF263, EGR1
	Motif 3	SVP, SPI1, IRF1
	Motif 4	SVP, ID1
	Motif 7	SVP, ZNF740, SP1, RREB1, KLF4/5, PLAG1, ZNF263, EGR1, SP3
	Motif 9	HOXD9
**Hypo intron**	Motif 1/2	Foxk1
**Hyper intron**	Motif 1/2	Foxk1
	Motif 3/4	SVP
	Motif 7	ZNF740, SP1, E2F4, ZNF263, KLF5, PPARγ, NR2C2, EGR1, SP3
	Motif 11	SP1, E2F4, ZNF263, KLF5, PPARγ, EGR1
